# Microbiome‐Based Colon Cancer Patient Stratification and Survival Analysis

**DOI:** 10.1002/cam4.70434

**Published:** 2024-11-21

**Authors:** Joshua Smyth, Julien Godet, Anisa Choudhary, Anubrata Das, Georgios V. Gkoutos, Animesh Acharjee

**Affiliations:** ^1^ College of Medical and Health, School of Medical Sciences, Cancer and Genomic Sciences University of Birmingham Birmingham UK; ^2^ Faculty of Pharmacy University of Strasbourg Strasbourg France; ^3^ ICube UMR 7357 CNRS, FMTS, University of Strasbourg Illkirch France; ^4^ Medical Information Department Clinical Research Methods Group, University Hospitals of Strasbourg Strasbourg France; ^5^ College of Medical and Health Institute of Clinical Sciences Birmingham UK; ^6^ Institute of Translational Medicine University Hospitals Birmingham NHS Foundation Trust Birmingham UK; ^7^ MRC Health Data Research UK (HDR) Birmingham UK; ^8^ Centre for Health Data Research University of Birmingham Birmingham UK

**Keywords:** clustering, machine learning, microbiome, personalised medicine, survival analysis

## Abstract

**Background:**

Colorectal cancer (CRC) is any cancer that starts in the colon or the rectum and presents a significant health concern. It is the third most diagnosed and the second deadliest cancer, with an estimated 153,020 new cases and 52,550 deaths in 2023. The severity of colon cancer may be attributed to its ability to avoid the host immune system and growth suppressors, its asymptomatic nature in the early stages, its association with a continually ageing population and unfavourable diet and obesity. The composition of the gut microbiome plays an important role in the development of CRC and presents as an important target in early detection and in predicting treatment outcomes in CRC. This study aims to identify microbiome‐specific derived clusters in CRC patients and conduct subsequent survival analysis using the specific microbiome features within clusters.

**Methods:**

Consensus clustering and feature selection, involving a Kruskal–Wallis test, a random forest and least absolute shrinkage and selection operator (LASSO) were applied resulting in the identification of differently expressed microbiomes between clusters. Lastly, survival analysis was performed on the selected features using Kaplan‐Meier curves and Cox regression. *K*‐means clustering, as selected using consensus clustering interpretation, presented three distinct clusters with clear differences in alpha and beta diversity and baseline clinical variables.

**Results:**

A total 1311 of the 1406 microbes were selected using the Kruskal Wallis and passed to the random forest and LASSO, which narrowed the dataset to 140 features. Following the survival analysis, eight microbiome species, namely *N4likevirus, Ambidensovirus, Synechococcus, Thermithiobacillus, Hydrocarboniphaga, Rhodovibrio, Gloeobacter* and *Candidatus Nitrosotenuis,* were selected as significant in clustering and survival.

**Conclusion:**

This study reveals the heterogeneity of the CRC microbiome and its effect on disease prognosis and necessitates further exploration of the biological mechanisms of these selected microbiomes as well further investigation of whether the approach depicted here is applicable to other cancer types.

## 1 Introduction

Colorectal cancer (CRC) is the collective term for all large bowel cancers, including the colon, the rectosigmoid junction and the rectum. Approximately two‐thirds of all colorectal cancers are accounted for by colon cancers, whilst cancers located at the rectosigmoid junction and rectum constitute the remaining one‐third [1]. CRC is the third most common cancer and the second most common cause of death due to cancer. It is estimated that there will be 3.2 million new colorectal cancer cases and 1.6 million deaths worldwide by 2040, compared to the 1.9 million new CRC cases and 930,000 deaths estimated in 2020 [2]. CRC spreads and proliferates partially due to its ability to avoid the host immune system, overcome growth suppressor mechanism and increase nutrient and blood supply exhibiting genetic instability. CRC is often asymptomatic in the early stages and therefore many cases are diagnosed once the cancer has already metastasized [3].

Although the causal mechanism of CRC is multifactorial, the microbiome plays a major role in CRC [4]. The gut microbiome refers to the community of microorganisms (e.g., fungi, bacteria and viruses) residing within the gut and is unique to every person. The gut microbiome consists of > 100 trillion microbes and is affected by factors such as genetics, exercise, diet, age and medication [5]. The gut microbiome has several beneficial and protective functions which include, assisting with digestion, immune response regulation, roles in metabolism and weight regulation and synthesising vitamins and amino acids [6]. However, an imbalance in the gut microbiome (dysbiosis) has been associated with conditions such as inflammatory bowel disease, atopic diseases, autism and potentially cancer [7]. Examples of microbes that have been linked to CRC progression include *Streptococcus bovis* , which is associated with an increase in Inflammatory cytokines, such as IL1β, IL‐8, TNF‐alpha and IL‐6; all of which have the potential to increase free radicals resulting in DNA alterations and subsequent cancer. As such, the microbiome may act as a biomarker enabling researchers to test at‐risk populations and monitor their progression. Interestingly, it is possible to manipulate the gut microbiota through treatment such as probiotics, prebiotics, diet and feacal microbiota transplantation [8] providing an opportunity to correct imbalances and improve conditions related to CRC. Machine learning and AI‐based methodologies are increasingly being utilised to determine the diagnostic potential of the microbiome [9].

Several studies have demonstrated the use of AI‐based methodologies for exploring the gut microbiome, for example, Chen et al. aimed to identify microbiome signatures for tumour subtype classification. This study processed data using the Kraken and SHOGUN methods and subsequently utilised various feature selection methods and four classification models. The SHOGUN method discovered that *Caballeronia* spp. was related to D‐tagatose biosynthesis and hepatocellular carcinoma initiation and progression. Moreover, *Gammaproteobacteria* exhibited distribution patterns in colorectal and pancreatic cancers [10].

A second study by Zhao et al., investigated alpha and beta diversity, bi‐clustering and a network‐based algorithm to investigate differences in microbiome culture between CRC patients and healthy controls. This study found a depletion of microbes in the CRC tumour and established that *Firmicutes* and *Bacteroides* were the most dominant phyla in CRC. Significant differences in microbial alpha diversity were observed between different subtypes of CRC, and the clustering consistently presented two distinct CRC microbial subtypes, showing heterogeneity within CRC patients [11]. Flemer et al. employed a least absolute shrinkage and selection operator (LASSO) and a random forest for feature selection for microbiome data of CRC patients and healthy controls to discover specific biomarkers of CRC [12]. Various oral taxa, such as *Streptococcus* spp. and *Prevotellas* spp., were disproportionately enriched in CRC patients compared to healthy controls [12].

Our study aims to stratify the CRC population by identifying microbiome‐specific clusters derived in CRC patients from public microbiome data sets. Identifying specific species or bacterial communities linked to CRC subtypes could enhance screening and diagnostic methods. Gaining insight into the underlying pathogenic mechanisms may pave the way for targeted interventions, including microbiome modulation and the development of pharmacological treatments for CRC prevention. Secondly, we aim to perform a validation analysis in the multiple CRC populations and identify key microbes associated with CRC. This process will provide us with information regarding the stability of microbiomes across multiple data sets. Lastly, we will investigate whether microbiome diversity impacts the survival of the cancer population.

## 2 Methods

### 2.1 Datasets

The data was collected from cBioportal using the Cancer Genome Atlas ‘TCGA, PanCancer Atlas’ dataset, specifically the microbiome data [13]. The Kraken method [14] was applied to 59,974 microbial genomes related to 32 cancer types. These genomes were initially downloaded from RepoPhlan [15] on June 14, 2023. The resulting dataset, prior to filtering, contained 71,782 genomes, including microbiome abundance data samples with 1406 operational taxonomic units (OTUs) comprising bacteria, archaea and viruses and 583 clinical variables. The number of samples and features for each of the data are shown in Table 1.

**TABLE 1:** List of the data used and corresponding sample sizes listed.

Cohort	Public data used	Sample and feature sizes	Reference
Discovery	TCGA	CRC (samples = 583, features = 1406)	[13]
Validation	Curated metagenomic data	CRC (samples = 435 features = 324)	[16]

### 2.2 Data Pre‐Processing

**FIGURE 1:**
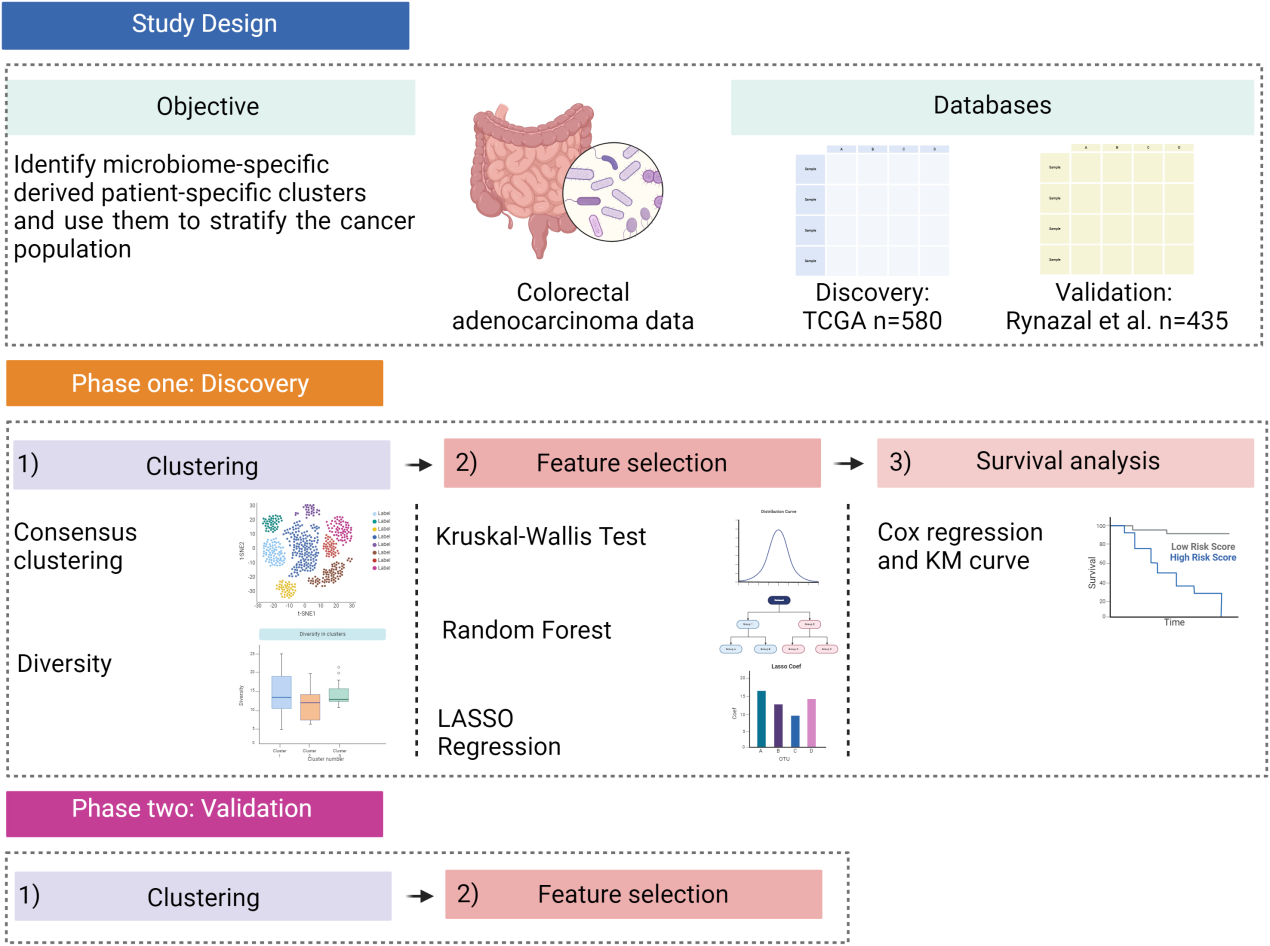
Flow chart of the overall study methodology depicting four key stages: Data pre‐processing, clustering, feature selection, and survival analysis.

There were no missing values in the microbiome dataset. Additionally, there was no zero variance present in the microbiome data implying that some OTUs are unchangeable throughout samples. Outliers were investigated both visually and computationally. To achieve this, multiple dimension reduction techniques were applied including principal component analysis (PCA) [17], principal coordinate analysis (PCoA) [18], *t*‐distributed stochastic neighbour embedding (tSNE) [19] and Uniform Manifold Approximation and Projection (UMAP) [20]. Finally, the microbiome data was normalised using range normalisation. Range normalisation involves subtracting the min (OTU) and dividing by the [max (OTU)−min (OTU)]. The workflow of the microbiome analysis is shown in Figure 1.

### 2.3 Unsupervised Machine Learning

#### 2.3.1 Optimal Number of Clusters

We used clustering to identify whether there were any distinct groups in the CRC patients’ microbiome and hence any heterogeneity. Four methods were utilised: Elbow plot, silhouette plot, gap statistic and clustree. An Elbow plot [21] was used on the pre‐processed microbiome data using an initial *K*‐means clustering and the width of the sum of squares methods, the sum of squared distances between data points and their assigned cluster centres across different numbers of clusters. To select the optimal number of clusters, the ‘elbow’ method was used, which minimally reduces cluster dissimilarity. Then a silhouette plot [22] was employed on the microbiome data using an initial *K*‐means clustering method. The average silhouette width measures the clusters’ similarity, so the number of clusters with the highest silhouette is selected. A gap statistic plot [23], relating to the within‐cluster dispersion of the data, was achieved using an initial *K*‐means clustering with the first max function and 10 iterations, and the number of clusters with the larger gap statistic was chosen. Lastly, a clustree [24] was used to show the allocation of clusters as the number of clusters increased. Clusters with similar samples with minimum interchangeability were selected (3 clusters) Table 2. We also performed a one‐way ANOVA and post hoc analysis with the clustered identified.

**TABLE 2:** Distributions of the clinical parameters across multiple clusters. ANOVA and Chi‐squared test were performed based on the quantitative or qualitative variables.

Clinical data	Cluster 1	Cluster 2	Cluster 3	ANOVA	Chi‐squared
Patient's number	159	314	97		
Diagnosed age	66.24	66.42	65.3	0.64	
Gender	50.31	51.91	57.73		0.49
TMB	16.14	13.89	8.52	0.13	
Progression free	26.05	24.21	20.91	0.09	
Months survived	29.14	27.42	22.02	0.03	
MSI status	5.17	4.22	2.9	0.08	
Mutation count	106.5	106	100	0.19	
Stage_I	34	46	19		0.15
Stage_II	15	13	6		0.36
Stage_III	3	18	1		0.12
Stage_IV	16	28	10		0.15

### 2.4 Consensus Clustering

Multiple clustering methods were applied, namely *K*‐means [25], hierarchical clustering [26], PAM clustering [27] and *C*‐means [28] along with the number of repeats and percentage resampling [29].

### 2.5 Microbiome Diversity

We used Alpha diversity to estimate the richness and distribution of microbiome species within a group and Beta diversity to estimate the species variance between groups [30]. Alpha diversity was calculated using the Shannon index, and beta diversity using the Manhattan index. A *t*‐test was also performed to find significant differences (*p* < 0.05) between alpha or beta diversity clusters.

### 2.6 Supervised Machine Learning

We used univariate feature selection which involves statistics analysis, multivariate feature selection which employs random forest [31] and LASSO [23]. These methods were used with the aim of selecting statistically different features between clusters.

### 2.7 Statistical Analysis

We used ANOVA to discover statistical differences between clusters. Before ANOVA, we used Bartlett test [32] to check equal variance, and the Shapiro–Wilk test [33] to check normality for each feature. In the case of non‐normal datasets, a Kruskal–Wallis test [29, 34] was applied across clusters. The resulting datasets were then used for the application of the Random Forest and LASSO methods.

### 2.8 Random Forest

A random forest algorithm was applied to discover the microbiome responsible for predicting the cluster number. The data was split into input variables, microbiome data and output cluster number. The *VarSelRF* algorithm was then used for feature selection. This algorithm determines the most important predictors by iteratively dropping a fraction of the least important variables from the input dataset. This process is repeated multiple times to evaluate the impact of each variable and identify the most important features. More specifically, the random forest had 500 trees, 300 iterations and a drop fraction of 20%. This feature selection process aids in the reduction of the model's complexity and improves performance by focusing on the most relevant variables.

### 2.9 Least Absolute Shrinkage and Selection Operator

LASSO is a linear regression technique that adds a penalty term (Lambda) to the ordinary least squares objective function, encouraging the model to shrink some coefficients of unimportant features to zero. The remaining feature with non‐zero coefficients is thus classed as important. The data was split into input and output variables and a train‐test split of 70%–30% and optimised using the minimum lambda value from the cross‐validation [23]. Cross‐validation was used to assess the model's performance on multiple subsections of the data, eventually selecting the optimal lambda value. The minimum Lambda attribute identified represents the minimum value that produces the best performance according to the specified evaluation metric. To further explore and visualise the differences in the relative abundance of the selected significant OTUs, a boxplot was created of each significant OTU.

### 2.10 Survival Analysis

The survival probability of each cluster was investigated by plotting each cluster using a Kaplan–Meier curve (KMC) [35] employing the time‐to‐event data and survival status. We used binary inputs based on above or below the mean or median abundance of the microbes. *p* < 0.05 was selected as significant in survival. The selected OTUs were combined with clinical data, and a Cox regression [36] was performed. A survival object was created using the time of the event and the survival status. A Cox proportional hazards model was then performed, outputting the hazard ratios of the significant OTUs and selected clinical data.

### 2.11 Validation Cohort

The validation cohort was extracted from Rynazal et al. using the curated MetagenomicData package [16]. Four datasets were utilised; Yachida et al. CRC microbiome data originated from Japan [37], Yu et al. from China [38], Vogtmann et al. from the USA [39] and Zeller et al. from France. We combined these datasets using the combat function that utilises the empirical Bayes method to adjust for the batch effects by estimating their distributions in the data to minimise the variation amongst batches while maintaining biologically relevant variability. The resulting dataset contained 435 samples and 324 microbiome species. We followed the same pre‐processing and clustering techniques that employed discovery methodology to detect any similar patterns.

## 3 Results

### 3.1 Baseline Statistics With Clinical Data

The microbiome dataset consisted of 583 samples and 1406 microbes. The average age of these 583 patients was 63.63, and gender was distributed evenly with a 52–48 male‐to‐female distribution. The data was stratified based on the CRC neoplasm disease stages (stages I–III, etc.). All categorical variables, disease‐free status, overall survival status and disease subtype, were deemed significantly different between disease stages, using an ANOVA and at a 0.05 significant level. The buffa hypoxia score and patient weight were not significantly different between disease stages. Diagnosis age, disease‐free months, progress‐free survival months and TMB significantly differed. The full table describing baseline patients’ demographics and clinical characteristics can be found in Table S1.

### 3.2 Clustering

Multiple dimension reduction methods (Figure S1) were used together with the clustering methods. The elbow of the elbow plot, the highest scores in silhouette, gap statistics and the stability in the clustree resulted in the selection of three clusters (Figure S2). Consensus clustering suggested *K*‐means was the optimal clustering technique. HC and PAM resulted in almost all samples assigned to cluster 1 and PAM only presenting two clear clusters. Moreover, the clustering parameters suggested that both *C*‐means and *K*‐means outperformed these methods, with notably higher Calinski Harabasz, Dunn, Davies Bouldin and Connectivity scores (Table S2). *K*‐means was chosen over *C*‐means (Figure S3). Additionally, we performed tSNE transformed data to cluster patients, there was an overlap between the clusters: 58 patients were common as cluster 1; 121 patients were cluster 2 and 31 patients were overlapping in cluster 3.

**FIGURE 2:**
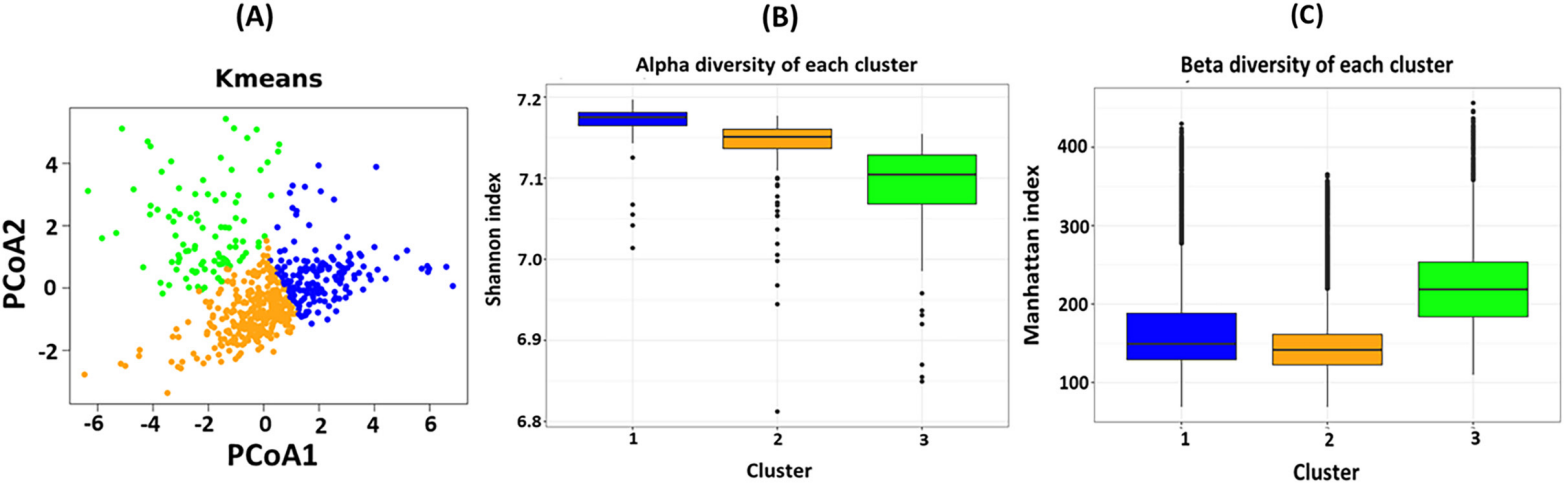
Microbiome data clustering results. (A) Final clustering plot based consensus clustering, A *K*‐means clustering plotted with Principal Coordinate Analysis (PCoA) (B) Alpha diversity using the Shannon index of the three clusters. (C) Beta diversity using the Manhattan index of the three clusters.

Figure 2A presents three distinct clusters of CRC patients alongside the clustering parameters. Figure 2B,C shows the characteristics of each cluster. Alpha diversity relates to within‐sample microbiome diversity and is measured using the Shannon index. Figure 2B presents each cluster with distinct variations in species richness and evenness, with cluster 3 differing substantially from clusters 1 and 2. Figure 2C presents clusters 1 and 2 with similarly low beta diversity, between cluster diversity, and cluster 3 with a relatively higher beta diversity. Collectively, these results present a strong indication of a clear CRC microbiome heterogeneity. In total, 570 samples are present, with 159 in Cluster 1, 314 in Cluster 2 and 97 in Cluster 3. Three variables were considered significantly different between clusters using an ANOVA or *t* test. Progress free survival months significantly differed between clusters 2 and 3 (*p* = 0.05). TMB nonsynonymous, the number of mutations present in the tumour, significantly differs between clusters 2 and 3 and clusters 1 and 3 (*p* = 0.09, *p* = 0.05, respectively). The CRC subtype significantly differed between all three clusters (*p* = 0.083), showing the heterogeneity of the CRC microbiome, and potentially showing a mechanism behind this differentially enriched microbiome with CRC patients. The baseline table stratified by cluster number is presented in Table S3.

### 3.3 Selecting Important Microbiota

Each sample was assigned its cluster number resulting from the optimal clustering method aiming to discover any significant OTU differences between clusters. A non‐parametric Kruskal–Wallis test identified 1368 OTUs (*p* < 0.05) differently expressed between clusters. To ensure a stringent selection, the *p*‐values were lowered to 0.001 resulting in 1311 OTUs which were then used as inputs to the Random Forest and LASSO algorithms. The confusion matrix, derived from the random forest analysis, resulted in a class error of 0.1949, 0.041 and 0.072 for clusters 1, 2 and 3, respectively, and an average out‐of‐bag error rate of 9.2426. By employing the varSelect algorithm subsequently, 10 features were selected. The LASSO algorithm had an accuracy of 0.906 and an AUC score of 0.882, with 130 OTUs. The resulting 140 differentially selected OTUs (10 from Random Forest and 130 from LASSO) were then used for survival analysis. Each significant OTUs abundance in each cluster was then represented using a boxplot (Figure S4).

### 3.4 Survival Analysis

**FIGURE 3:**
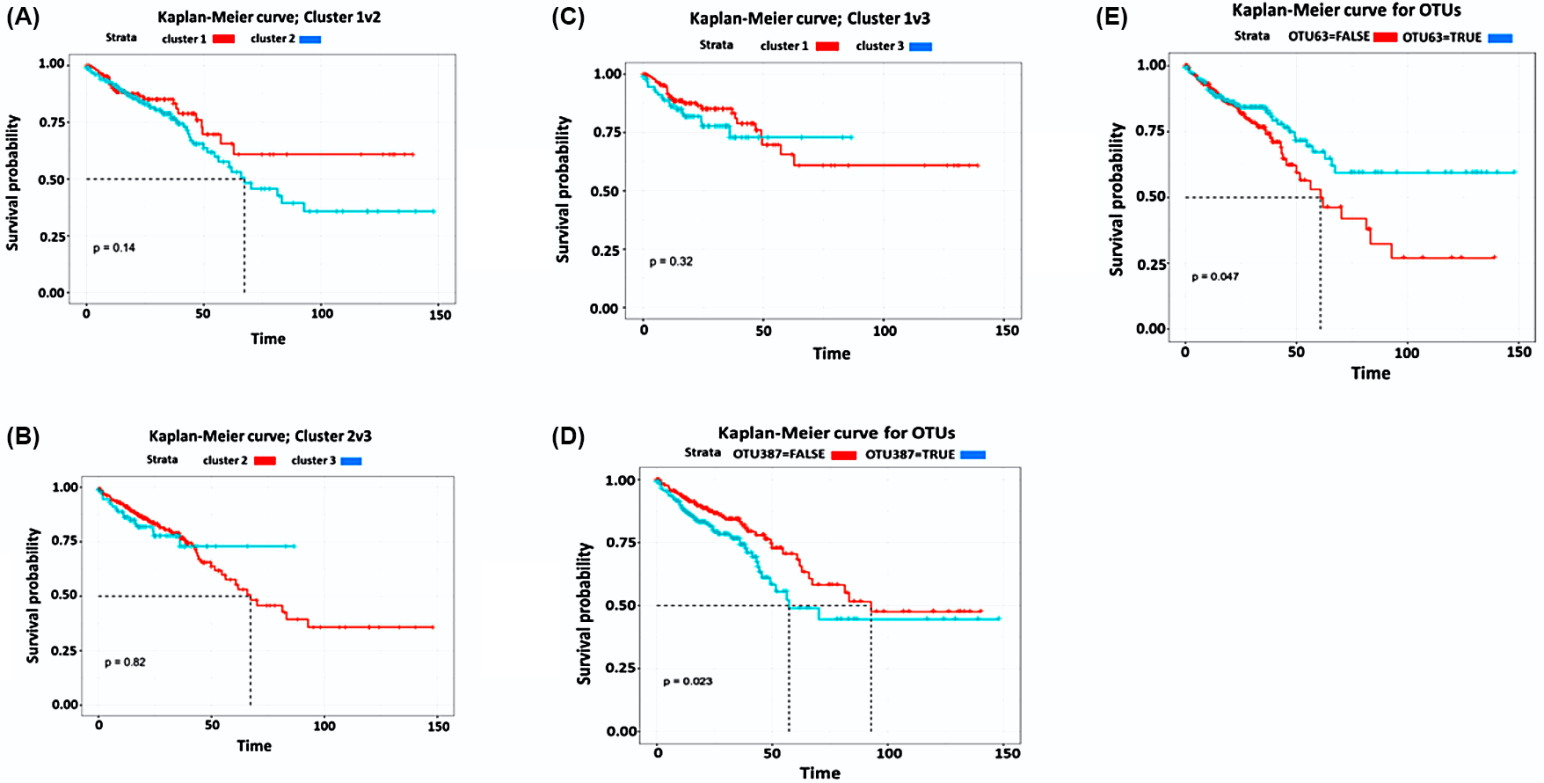
Kaplan–Meier curves of (A) Cluster 1 versus 2, (B) Cluster 2 versus 3, and (C) Cluster 1 versus 3. The *p*‐values generated from the log‐rank test between the two groups. (D) operational taxonomic unit (OTU) using two groups more than mean values. (E) OTU using two groups as more than the median values.

The KMC represents survival status over time with cluster 2, which was represented by a worse prognosis than clusters 1 and 3. This was plotted using microbiome data, cluster number, survival status and time‐to‐event, possibly showing that differential microbiome with CRC patients can affect prognosis (Figure 3A–C). To understand the potential role of each microbiome in survival status, each microbiome sample was changed to determine whether it was above or below the mean or median concentration. Two significant examples (*p* < 0.05) of KMC from both mean‐binarized and median‐binarized are presented in Figure 3D,E. All Kaplan–Meier curves are provided in Figures S5 and S6. We reiterated the ANOVA analysis conducted earlier between the three clusters, on the data available from the Kaplan–Meier curves. The clusters showed a statistical difference between means (*p* = 0.007), with the one‐way ANOVA test. To understand which clusters were different, we conducted a post hoc Tukey's test and found that clusters 1 and 3 differed significantly (*p* = 0.041). Thus, demonstrating the difference in the microbiome in the clusters impacts the majority of the population. The mean‐binarized selected six OTUs, while the median‐binarized OTUs selected five OTUs with three OTUs overlapping. Thus, eight OTUs were selected, namely *N4likevirus*, *Ambidensovirus*, *Synechococcus*, *Thermithiobacillus*, *Hydrocarboniphaga*, *Rhodovibrio*, *Gloeobacter* and *Candidatus Nitrosotenuis*.

The hazard ratios of the resulting eight OTUs along with the clinical data derived using a Cox regression varied for each OTUs denoted by a large 95% confidence interval. A*mbidensovirus* was the OTU with the most consistently high hazard ratio. *Hydrocarboniphaga* was the OTU with the most consistently low hazard ratio. Clinical data, for example, disease‐free months and patient weight were found reduced hazard ratios (Table S4).

### 3.5 Validation

The dataset used for validation was collected containing multiple microbiome datasets of CRC patients [16] These datasets were then combined using the combat function to create the validation dataset (Figure S7). Three similarly distinct clusters were discovered using *K*‐means clustering (Figure S8) and (Table S5), although a similar alpha diversity between clusters was observed, the beta diversity was altered significantly (Figure S9). Furthermore, following a feature selection process, one genus, *Haemophilus*, out of the 75 selected by the LASSO and random forest in the discovery dataset, was found to be overlapping.

## 4 Discussion

We identified three distinct clusters with marked alpha and beta diversity variation and baseline features, such as CRC subtype and tumour mutational burden (TMB). Following a survival analysis, eight microbiome species were selected as significant, namely *N4likevirus*, *Ambidensovirus*, *Synechococcus*, *Thermithiobacillus*, *Hydrocarboniphaga*, *Rhodovibrio*, *Gloeobacter* and *Candidatus Nitrosotenuis*. Furthermore, the feature selection process revealed an overlap in one microbiome at the genus level, *Haemophilus*. These results delineate the diverse nature of the microbiome in CRC and its detrimental impact on the prognosis of the disease.

Out of the eight microbiome species selected, only one, *N4likevirus* has been previously associated with carcinogenesis in the literature with Gihawi et al. demonstrating *N4likevirus* as one of the top 10 features in distinguishing adrenocortical carcinoma [40] The genus *N4likevirus* possesses a virion‐associated RNA polymerase, thus being involved in DNA metabolism, host interaction, replication, virion structure, lysis and packaging [41] However, the potential relevance of *N4likvirus* in adrenocortical carcinoma remains unclear [40].

*Ambidensovirus* is linked to DNA helicase and, thus, DNA replication [42], *Synechococcus*’*s* has been correlated to cell apoptosis via the JNK and p38 MAPK signalling pathways in human colon carcinoma cells [43] *Thermithiobacillus* has been shown as oxidising inorganic sulphur compounds to gain energy. While studies have examined its contribution to environmental processes, no current literature exists demonstrating its relation to CRC prognosis [44], *Hydrocarboniphaga* function in hydrocarbon degradation to convert into fatty acids, which can then be metabolised to produce energy [45], *Rhodovibrio* can fix nitrogen and aid the metabolisms of organic molecules, including sugars, organic acids, amino acids and fatty acids [46] *Gloeobacter* is involved in photosynthesis, nitrogen fixation and lipid metabolism, including fatty acid synthesis, modification and storage [47], *Candidatus nitrosotenuis* is mainly known for its role in the nitrogen cycle and carbon metabolism; thus, its relation to CRC remains unknown [48].

Limited studies show a clear correlation between the specific microbiome mentioned above and CRC, some; however, have shown functioning linked to pathways involved in cancer progression, including DNA replication, cell death and energy production. Nevertheless, as no studies to date have reported on the relevance of these microbes in either CRC or carcinogenesis in general, it is unclear currently how informative these microbes might be in CRC survival without further research. Further research could focus on isolating these specific microbes and introducing them into knock‐out mice with CRC and determining how CRC survival is affected in mice with and without these microbes prospectively.

The CRC microbiome heterogeneity illustrated could be the result of several factors including lifestyle factors, such as diet, medication. Multiple cancer development pathways can affect the microbiome composition. For example, there is a distinct difference in the microbiome between left and right‐sided CRC including molecular differences such as microsatellite instability, mucinous type and certain pathway activation. The tumour microenvironment can also affect the microbiome, pH, oxygen levels and nutrients. Different cancer treatment types will also affect the microbiome and chemotherapy, radiotherapy and immunotherapy can significantly affect normal microbial balance. Finally, microbiome variability directly results in differences in immune response, digestion and synthesis of by‐products which in turn further alters the host environment [49].

Rynazal et al. utilised similar methods like *K*‐means clustering, PCA, etc. using related methods and also demonstrated heterogeneity across patients and further identified plausible CRC biomarkers. Machine learning and AI models are rapidly becoming essential tools for uncovering patterns in microbiome data sets [50, 51].

It is important to note that while microbes may be protective and prevent against carcinogenesis, microbes can also stimulate carcinogenesis by synthesising carcinogenic products, elevating inflammation, mutations and shaping the cell cycle and signalling pathways. The ability to promote and initiate cancer affects survival risk [52] One interesting study outlined the effect of the intratumorally microbiota on CRC heterogeneity where bacterial‐infected CRC cells showed altered gene expression related to infection response, inflammation, hypoxia, cancer cell progression and metastasis [53]. Debelius et al. aimed to explore the relationship between microbiome and survival in patients with late‐stage CRC undergoing resection for primary adenocarcinoma. They found tumour tissue had a higher concentration of *Fusobacteria*, *Porphyromonas*, *Granulicatella* and *Campylobacter*, whilst a lower relative abundance of *Blautia* and *Ruminococcus*. Using a robust PCA, clear differences in the microbiome between short‐term and long‐term survivors were identified. Lastly, *Fusobacterium*, *Parvimonas*, *Porphyromonas*, *Gemella* and *Dialster* were depicted as related to long‐term survival, while *Escherichia* and *Shigella* were linked to short‐term survival [54].

Microbial metabolomics has been used in recent studies on CRC, focusing on amino acids, lipids, ketone bodies, carbohydrates and short‐chain fatty acid microbial metabolites [55]. These studies reveal distinct gut‐microbiome‐derived phenotypes in early‐onset CRC, with microbial metabolites such as butyric acid‐producing bacteria, *Fusobacterium nucleatum* , *Lachnospiraceae bacterium GAM79* and *Collinsella aerofaciens* [56]. Metabolic comparisons of patients with CRC at different anticancer treatment stages reveal fatty acids like N‐phenylacetylglycine upregulated, ketone bodies like 4‐hydroxy phenylacetate, acetate and carboxylic acids like succinate. Circulating amino acid levels and CRC risk are also explored in the European Prospective Investigation into Cancer and Nutrition and UK Biobank Cohorts [57]. These studies provide unique insights into how specific metabolites and microbes are altered in CRC, providing potential biomarkers [58] and therapeutic targets and diagnostics [9].

Our approach has many potential limitations. Firstly, only one dataset was used for discovery, and the validation dataset had a small sample size, thereby reducing the results’ generalisability. Additionally, although 13 outliers were removed using dimension reduction, range normalisation might not have been the most suitable method due to its sensitivity to extreme values. Moreover, the small sample size reduces the statistical power and diversity, especially when data are further split into groups for statistical analysis. Furthermore, the distribution of samples across clusters was imbalanced, which can affect the accuracy and reliability of the findings. The validation results are limited since a small, missing survival information of the patients, albeit varied, dataset was used and hence replicating our results across multiple populations would strengthen their robustness. The biological mechanism underlying the microbiome and CRC relations necessitates further investigations, including better insights into the metabolic pathways, genes and proteins linked with the selected OTUs. Furthermore, longitudinal studies would be required to understand the microbiome changes over time as well as the effect of these changes on disease progression and survival. Integrating microbiome data with other types of omics data, such as genomics, transcriptomics, or metabolomics, would be essential for understanding the molecular landscape of CRC and would aid the identification of therapeutic targets. It is unclear how the patients were treated, which is important since different treatments could affect the microbiome [59]. Changes in the microbiome can occur at various cancer disease stages, a factor that we did not account for [60].

## 5 Conclusion

Research assessing the correlation between microbiome and survival, especially across CRC patients, is limited. CRC remains a significant global health concern as the second leading cause of cancer‐related deaths and the third most common cancer. We found multiple species linked with the CRC and survival. *N4‐like* viruses, *Ambidensoviruses*, *Synechococcus cyanobacteria*, *Thermithiobacillus*, *Hydrocarboniphaga bacteria*, *Rhodovibrio*, *Gloeobacter and Candida nitrosotenuis* are eight bacteria that have been linked to CRC. Some of the species are related to the contamination (for example: *Cyanobacteria*) through water or dietary sources. *Gloeobacter* alterations in microbiota composition may lead to CRC‐related dysbiosis. *Candida Nitrosotenuis*, an ammonia‐oxidising archaea, plays a role in nitrogen cycling. Although there are several studies investigating the association between the microbiome and cancer, they are primarily aimed at colon cancer patients’ stratification. Using clustering, feature selection and survival analysis, we identified eight microbiome species, statistically significantly differentially expressed across different clusters, and statistically significant within survival analysis models. Future research is required into the potential mechanisms underlying these associations. Nevertheless, our work reveals the heterogeneity of CRC patients’ microbiome highlighting its potential effect on prognosis, as well as its potential as a diagnostic and therapeutic tool.

## Author Contributions

**Joshua Smyth:** data curation (equal), methodology (equal), software (equal), writing – original draft (equal), writing – review and editing (equal). **Julien Godet:** formal analysis (supporting), methodology (supporting), software (supporting), writing – original draft (equal), writing – review and editing (equal). **Anisa Choudhary:** data curation (equal), resources (equal), writing – original draft (equal), writing – review and editing (equal). **Georgios V. Gkoutos:** funding acquisition (equal), writing – original draft (equal), writing – review and editing (equal). **Animesh Acharjee:** conceptualization (lead), funding acquisition (lead), methodology (lead), project administration (lead), resources (lead), software (equal), supervision (lead), writing – original draft (equal), writing – review and editing (equal).

## Ethics Statement

The authors have nothing to report.

## Consent

The authors have nothing to report.

## Conflicts of Interest

The authors declare no conflicts of interest.

## Supporting information


Data S1‐S4.

## Data Availability

All the data used in this study are freely and publicly available. R scripts and data sets are available here: 10.6084/m9.figshare.26046184.
