# Supplementary material for: Microbiome‐Based Colon Cancer Patient Stratification and Survival Analysis
Source: Cancer Med. 2024 Nov 21;13(22):e70434. doi: 10.1002/cam4.70434 (PMC11579663; doi:10.1002/cam4.70434)
Supplement: Supplementary file 1 — Data S1‐S4. [file CAM4-13-e70434-s001.docx]

**SUPPLEMENTARY MATERIAL**

**Supplementary Figure 1:** Four dimension reduction techniques are used to visualise the outliers in the discovery dataset. Overall, 13 samples were selected to be divided from the main group and thus removed from the dataset.

**
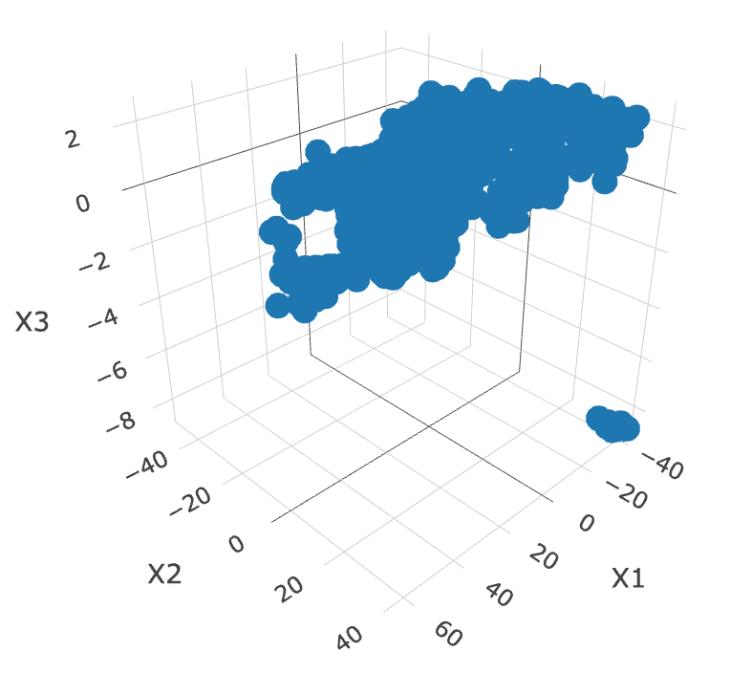

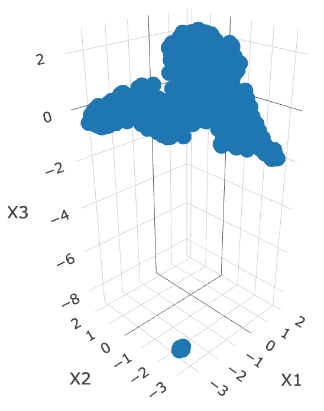
**
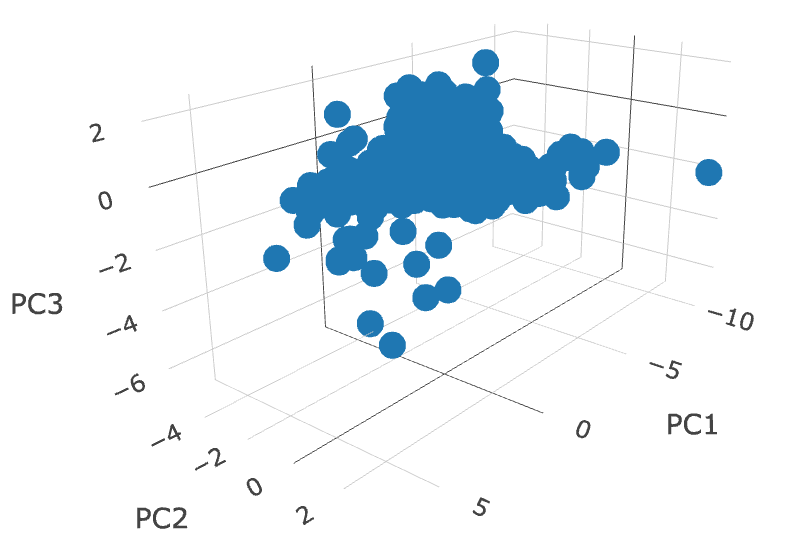
UMAP tSNE PCA


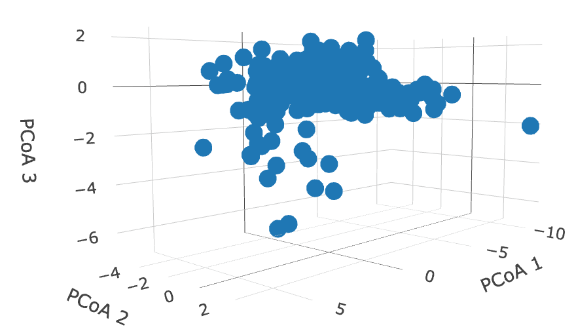
PCoA

**Supplementary Figure 2:** Four methods of selecting the optimal number of clusters for the discovery dataset were utilised. Three clusters were chosen based on the elbow plot's elbow, maximal scores in silhouette and gap statistic and stability in the clustree.


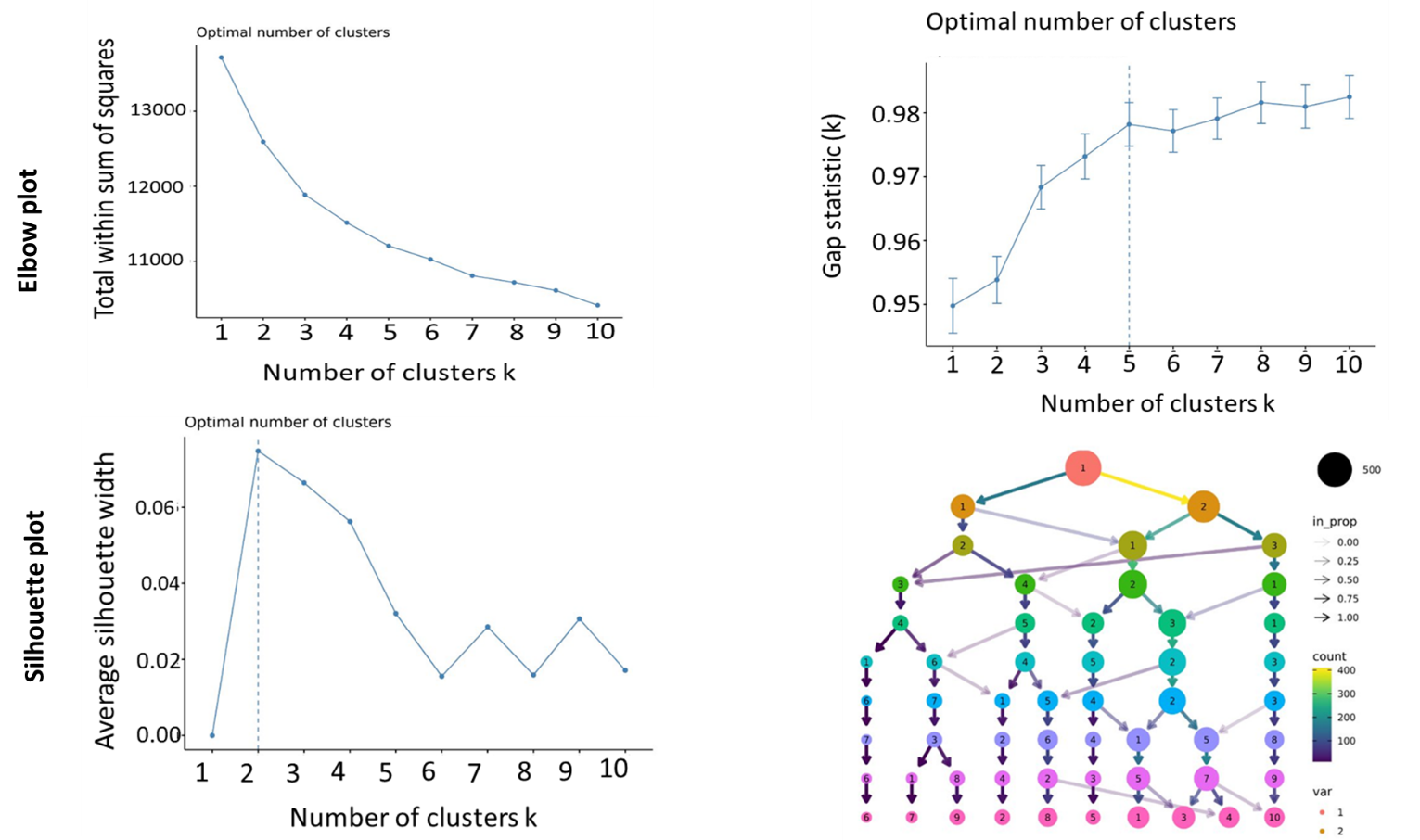


**Supplementary Figure 3:** The consensus clustering of the other three clustering techniques for discovery is plotted with a PCoA and coloured on cluster numbers. K-means was the optimal method; HC resulted in almost all samples in cluster 1, PAM only presented two clusters, and K-means performed better in clustering parameters.

**
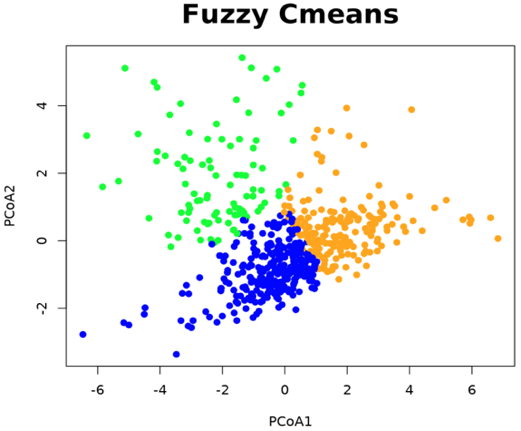

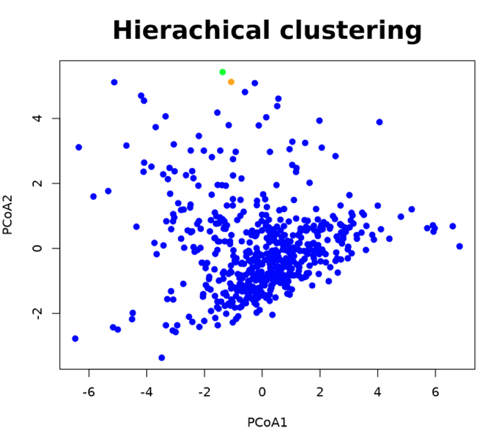

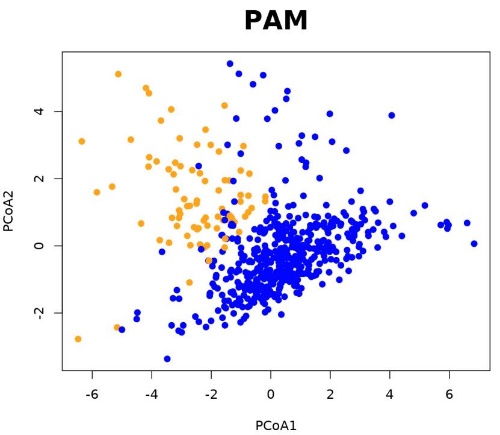
**

**Supplementary Figure 4:** Kaplan-Meier curve of the six significant OTUs after mean-binarisation. The lines are coloured on whether the sample is above or below the mean or median for that OTU. The lines are coloured on cluster numbers, and the p-value represents a t-test between the two groups.


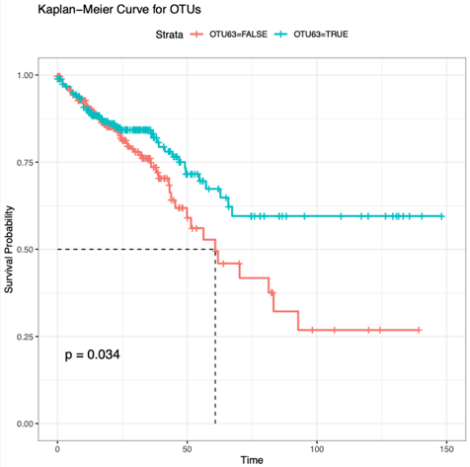


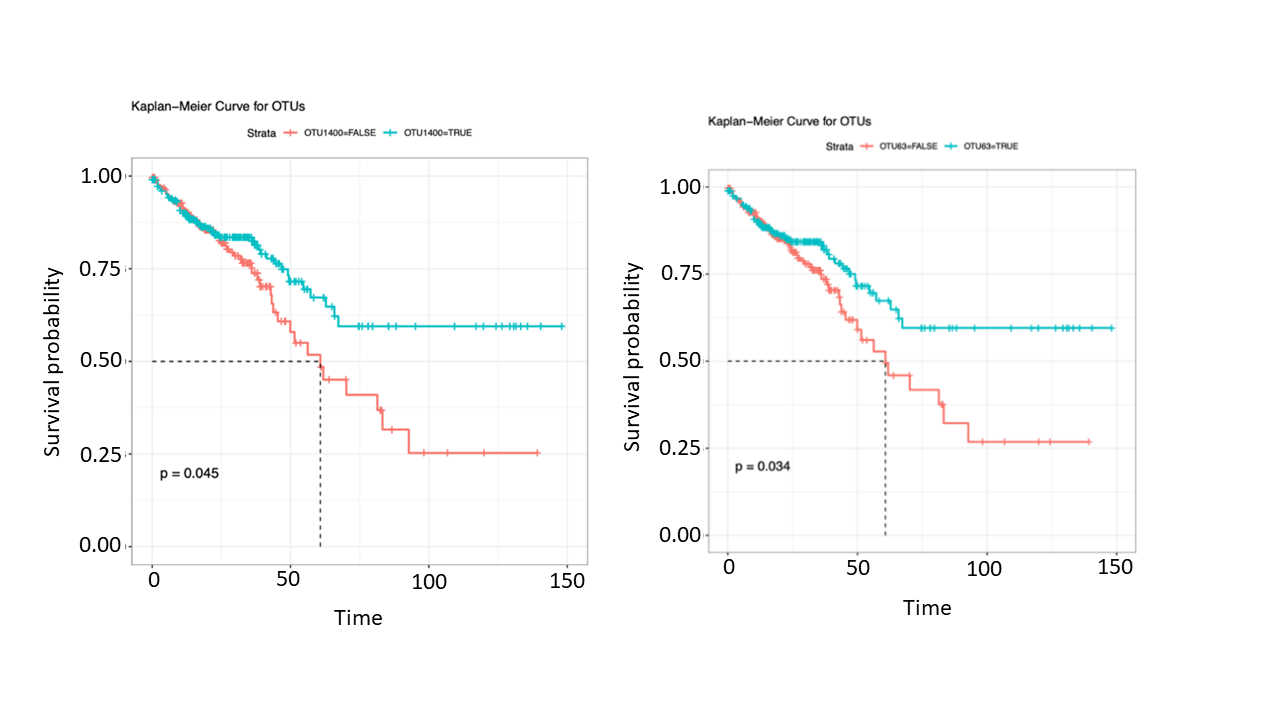


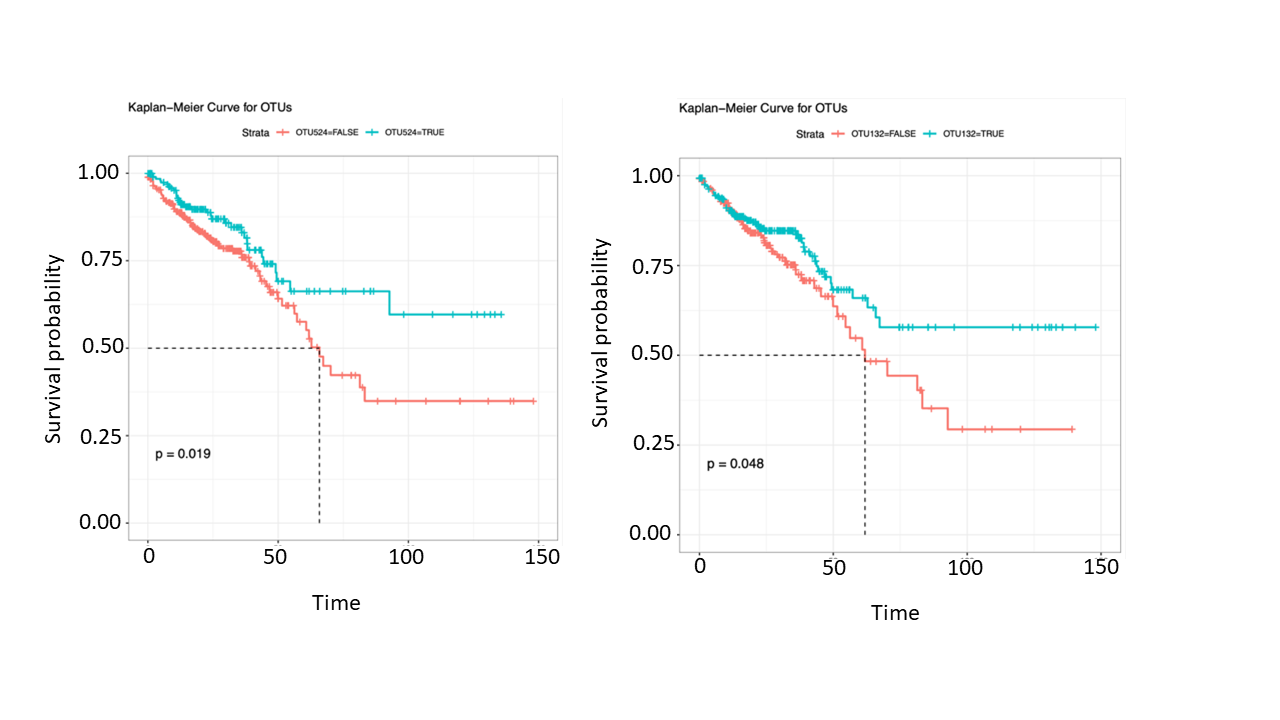


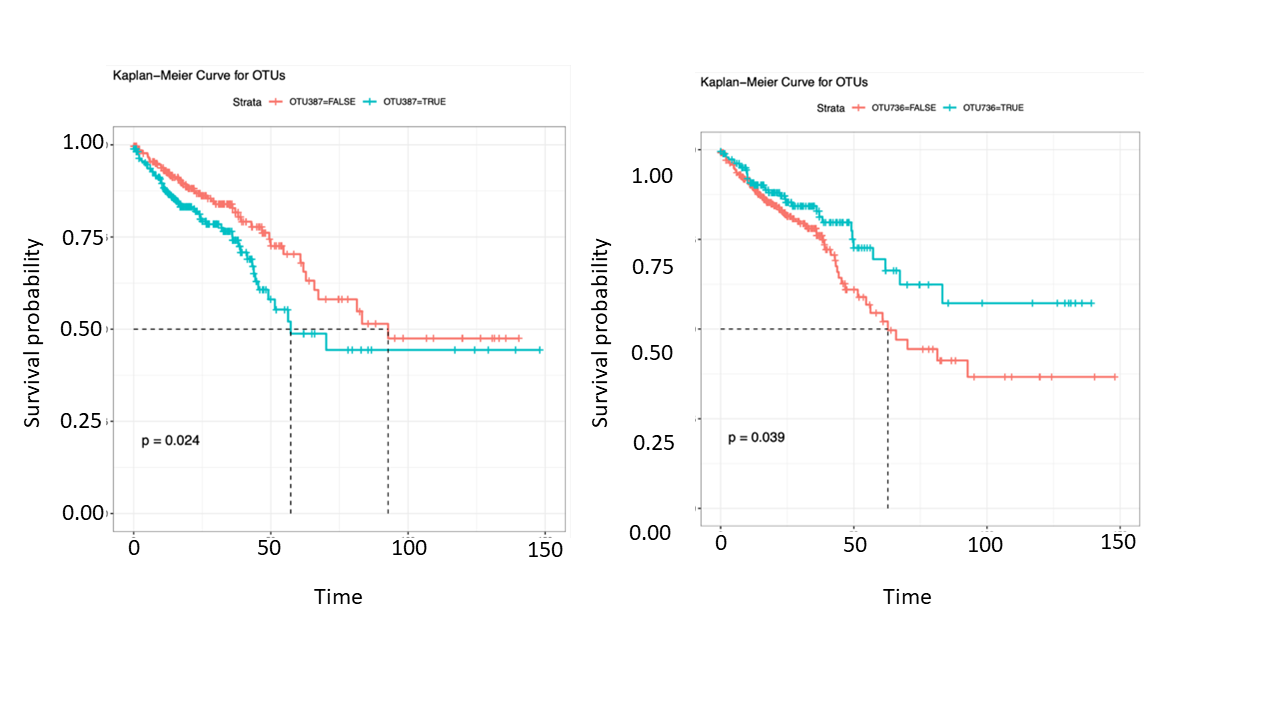


**Supplementary Figure 5:** Kaplan-Meier curve of the five significant OTUs after median-binarisation. The lines are coloured on whether the sample is above or below the mean or median for that OTU. The lines are coloured on cluster numbers, and the p-value represents a t-test between the two groups.


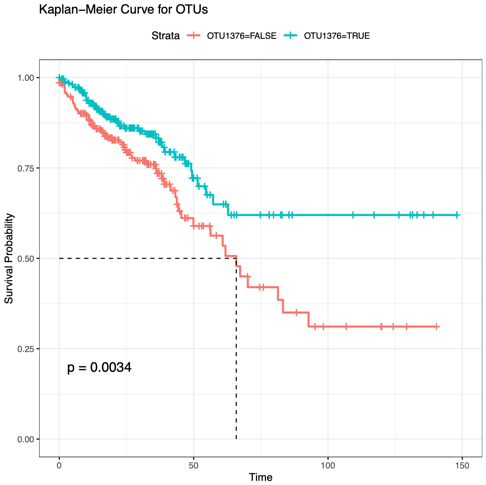

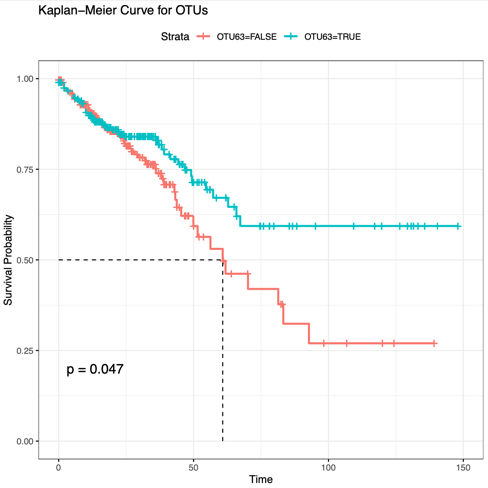

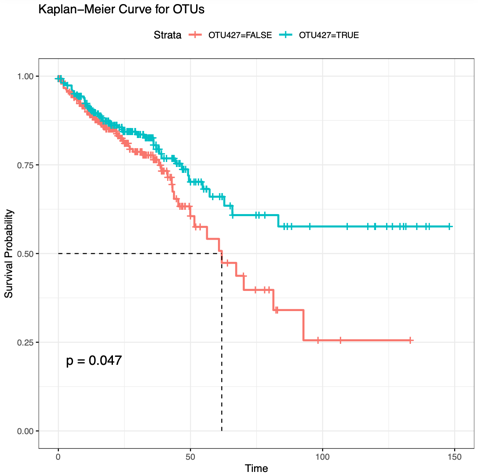

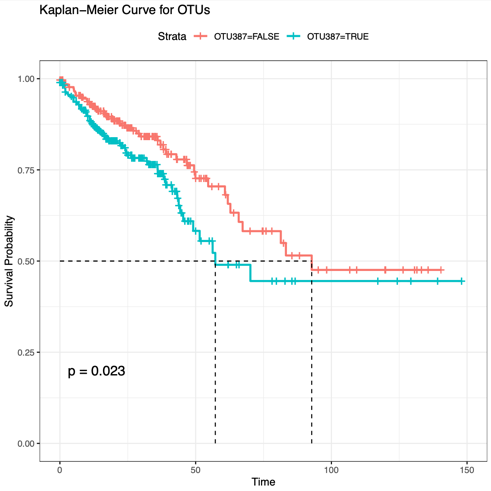


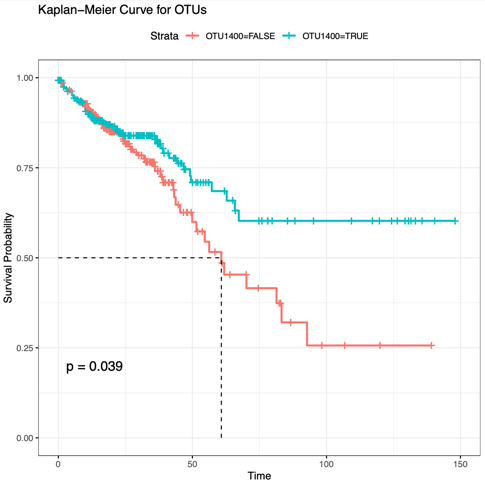


**Supplementary Figure 6:** A PCA coloured by the four datasets in the validation dataset before using a combat function to visualise the dissimilarity in their microbiome and after to illustrate the combat function's effect and hence the confounding factors' influence.

**
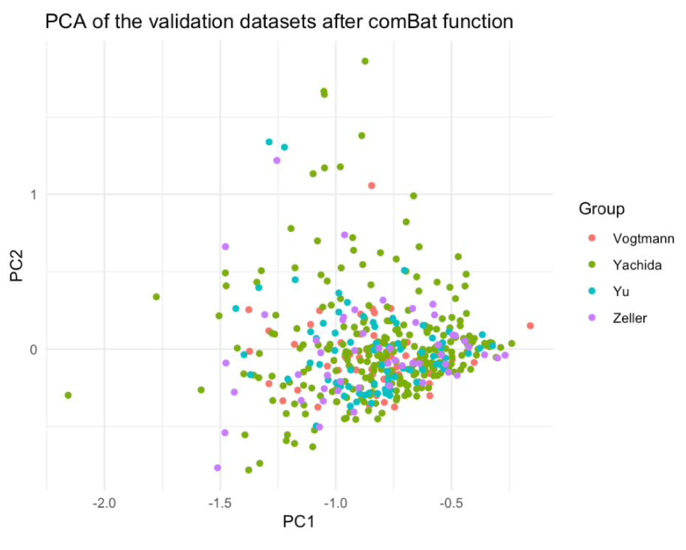
**


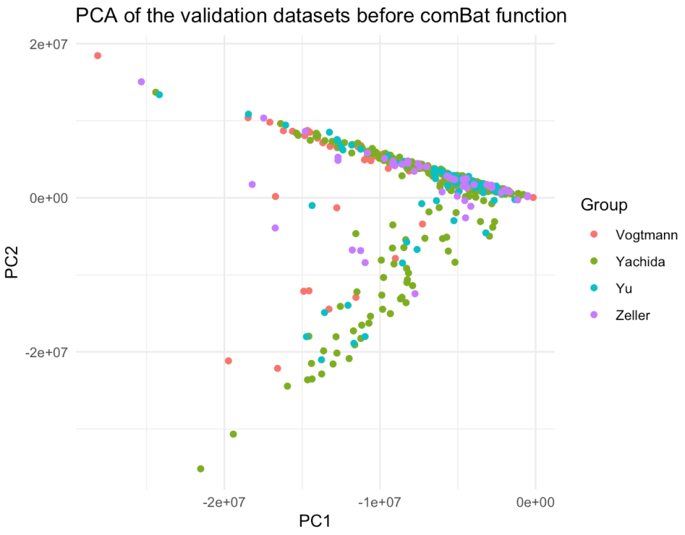


**
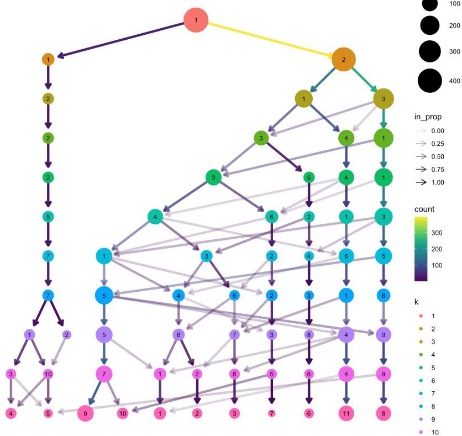
Supplementary Figure 7:** Four methods of selecting the optimal number of clusters for the validation dataset were utilised. Three clusters were chosen based on the elbow plot's elbow, maximal scores in silhouette and gap statistic and stability in the clustree.


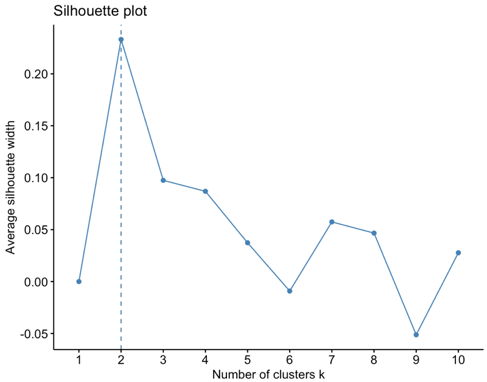

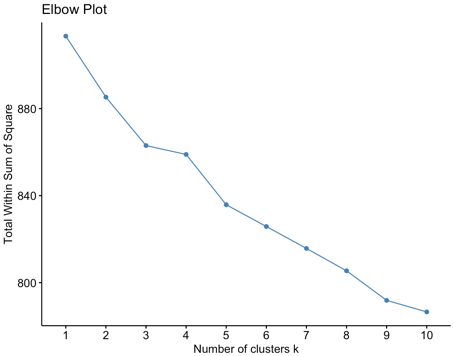


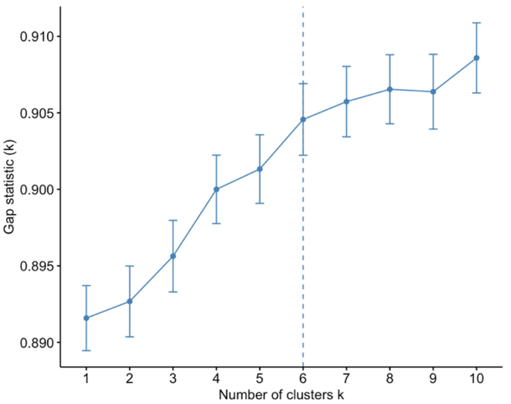


**
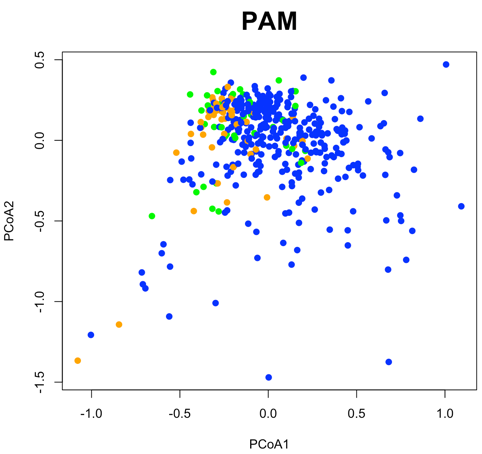
Supplementary Figure 8:** The consensus clustering of the four clustering techniques for validation is plotted with a PCoA and coloured on cluster number. K-means was the optimal method; HC, PAM and C-means resulted in almost all samples in cluster 1, and K-means performed better in clustering parameters.

**
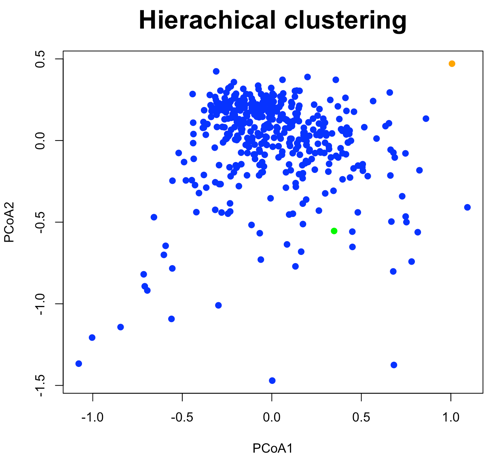

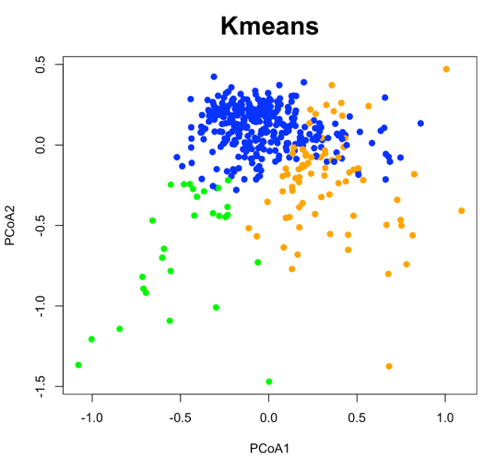

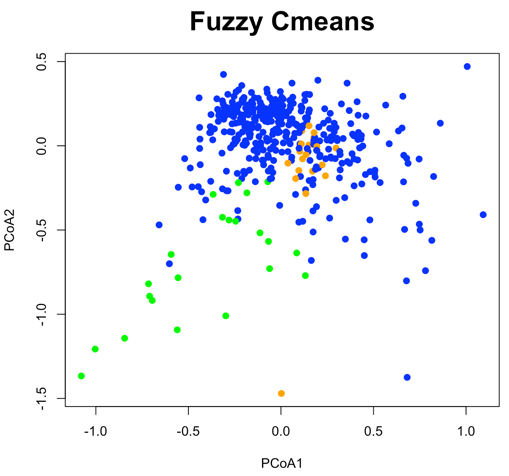
**

**
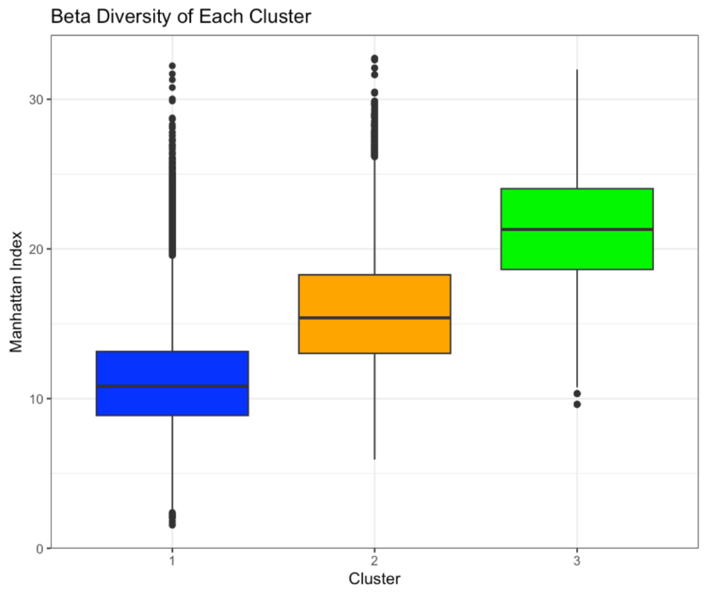
Supplementary Figure 9:** A boxplot showing the microbiome diversity of the clusters in the validation dataset, coloured by cluster number. Alpha diversity is calculated using the Shannon index, and beta diversity with the Manhattan index. Although alpha diversity between clusters was similar, beta diversity changed significantly.


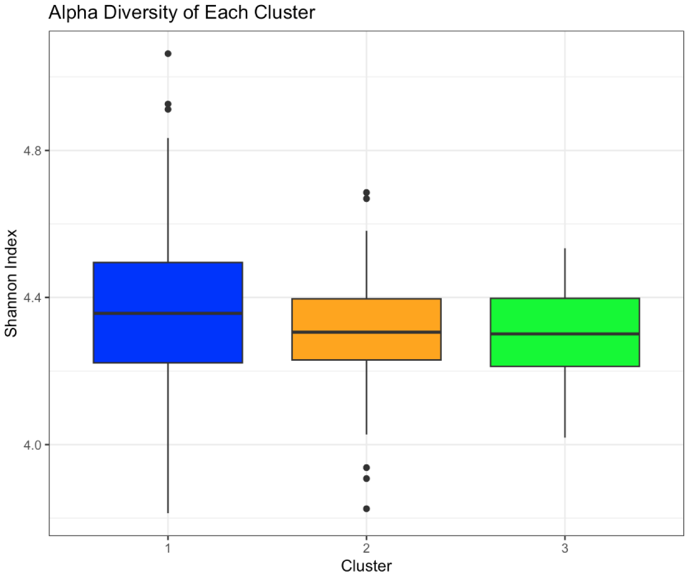


**Supplementary Table 1:** A baseline table of both categorical and numerical features stratified by disease stage. All categorical variables were deemed significantly different between disease stages, using an ANOVA (P<0.05), and only the buffa hypoxia score and patient weight were not significantly different. Diagnosis age, disease-free months, progress-free survival months and TMB also significantly differed.

| Categorical features | Overall | Missing | Stage I | Stage II | Stage III | Stage IV |
| --- | --- | --- | --- | --- | --- | --- |
| N | 583 | 12 | 103 | 215 | 168 | 85 |
| Disease_Free_Status (%) |  |  |  |  |  | 0 |
| 0:DiseaseFree | 189 | 7 | 48 | 79 | 55 | 0 |
| 1:Recurred/Progressed | 27 | 1 | 3 | 13 | 10 | 0 |
| Missing | 367 | 4 | 52 | 123 | 103 | 85 |
| Overall_Survival_Status (%) | |  |  | 0 |  | 0 |
| 0:LIVING | 463 | 7 | 96 | 181 | 130 | 49 |
| 1:DECEASED | 119 | 5 | 7 | 33 | 38 | 36 |
| Missing | 1 | 0 | 0 | 1 | 0 | 0 |
| Sex = Male (%) | 308 | 7 | 57 | 114 | 82 | 48 |
| Subtype (%) |  |  |  |  |  | 0 |
| COAD_CIN | 222 | 6 | 25 | 72 | 77 | 42 |
| COAD_GS | 48 | 0 | 16 | 19 | 10 | 3 |
| COAD_MSI | 60 | 1 | 13 | 33 | 11 | 2 |
| COAD_MOLE | 6 | 0 | 0 | 6 | 0 | 0 |
| Missing | 131 | 1 | 27 | 50 | 31 | 22 |
| READ_CIN | 100 | 3 | 17 | 28 | 37 | 15 |
| READ_GS | 9 | 1 | 3 | 3 | 1 | 1 |
| READ_MSI | 3 | 0 | 0 | 3 | 0 | 0 |
| READ_POLE | 4 | 0 | 2 | 1 | 1 | 0 |

| Numerical features | Stage I | Stage II | Stage III | Stage IV |
| --- | --- | --- | --- | --- |
| Diagnosis Age | 116.79 | 277.81 | 256.36 | 176.24 |
| Buffa Hypoxia Score | 29.36 | 79.7 | 63.87 | 36.73 |
| Disease Free Months | 28.97 | 124.13 | 136.71 | 0 |
| Progress Free Survival Months | 38.31 | 97.77 | 93.76 | 40.35 |
| TMB nonsynonymous | 16.31 | 112.23 | 39.44 | 11.5 |
| Patient Weight | 172.53 | 286.83 | 331.73 | 258.57 |
| Numerical features | Stage I | Stage II | Stage III | Stage IV |
| Diagnosis Age | 116.79 | 277.81 | 256.36 | 176.24 |
| Buffa Hypoxia Score | 29.36 | 79.7 | 63.87 | 36.73 |
| Disease Free Months | 28.97 | 124.13 | 136.71 | 0 |
| Progress Free Survival Months | 38.31 | 97.77 | 93.76 | 40.35 |
| TMB nonsynonymous | 16.31 | 112.23 | 39.44 | 11.5 |
| Patient Weight | 172.53 | 286.83 | 331.73 | 258.57 |

**Supplementary Table 2:** The clustering parameters show the clustering efficiency of each of the four clustering methods. C-means and K-means have notably higher Calinski Harabasz, Dunn, Davies Bouldin, and connectivity scores. K-means were chosen over C-means based on personal preference and a better understanding.

| Algorithms | Calinski Harabasz | Dunn | Gamma | C index | Davies Bouldin | SD | S dbw | Silhouette | Compactness | Connectivity |
| --- | --- | --- | --- | --- | --- | --- | --- | --- | --- | --- |
| HC | 4.515 | 0.738 | 0.977 | 0.058 | 0.451 | 1.415 | Inf | 0.389 | 2.971 | 5.858 |
| PAM | 4.475 | 0.097 | 0.04 | 0.474 | 5.183 | 6.467 | Inf | 0.012 | 2.978 | 277.817 |
| KM | 6.911 | 0.218 | 0.526 | 0.231 | 4.701 | 7.857 | Inf | 0.097 | 2.947 | 195.722 |
| C-means | 6.987 | 0.19 | 0.241 | 0.358 | 4.893 | 9.167 | Inf | 0.037 | 2.955 | 248.888 |

**Supplementary Table 3:** A baseline table of both categorical and numerical features stratified by cluster number. Progress Free Survival Months significantly differed between clusters 2 and 3 (p<0.05). TMB nonsynonymous significantly differs between clusters 2 and 3 and 1 and 3. The colorectal cancer subtype significantly differed between all three clusters.

| Numerical features | Stratified on cluster | | |
| --- | --- | --- | --- |
|  | 1 | 2 | 3 |
| Diagnosis Age | 66.24 | 66.42 | 65.3 |
| Buffa Hypoxia | 18.74 | 16.2 | 18.02 |
| Disease Free Months | 29.46 | 31.79 | 29.12 |
| Progress Free Survival Months | 26.05 | 24.21 | 20.91 |
| TMB nonsynonymous | 16.14 | 13.89 | 8.52 |
| Patient Weight | 79.49 | 80.1 | 80.38 |

| Categorical Features | Stratified by cluster | | | |
| --- | --- | --- | --- | --- |
|  | Overall | 1 | 2 | 3 |
| n | 570 | 159 | 314 | 97 |
| Disease_free_status (%) |  |  |  |  |
| 0:DiseaseFree | 184 (32.3) | 57 (35.8) | 97 (30.9) | 30 (30.9) |
| 1:Recurred/Progressed | 27 (4.7) | 9 (5.7) | 17 (5.4) | 1 (1.0) |
| Missing | 359 (63.0) | 93 (58.5) | 200 (63.7) | 66 (68.0) |
| Overall_Surival_Status (%) |  |  |  |  |
| 0:Living | 454 (79.6) | 132 (83.0) | 243 (77.4) | 79 (81.4) |
| 1:Deceased | 115 (20.2) | 27 (17.0) | 70 (22.3) | 18 (18.6) |
| Missing | 1 (0.2) | 0 (0.0) | 1 (0.3) | 0 (0.0) |
| Sex = Male (%) | 299 (38.8) | 80 (50.3) | 163 (51.9) | 56 (57.7) |
| Subtype (%) |  |  |  |  |
| COAD_CIN | 221 (38.8) | 57 (35.8) | 131 (41.7) | 33 (34.0) |
| COAD_GS | 47 (8.2) | 15 (9.4) | 28 (8.9) | 4 (4.1) |
| COAD_MSI | 59 (10.4) | 19 (11.9) | 33 (10.5) | 7 (7.2) |
| COAD_POLE | 6 (1.1) | 3 (1.9) | 3 (1.0) | 0 (0.0) |
| Missing | 123 (21.6) | 31 (19.5) | 68 (21.7) | 24 (24.7) |
| READ_CIN | 98 (17.2) | 31 (19.5) | 41 (13.1) | 26 (26.8) |
| READ_GS | 9 (1.6) | 1 (0.6) | 7 (2.2) | 1 (1.0) |
| READ_MSE | 3 (0.5) | 2 (1.3) | 0 (0.0) | 1 (1.0) |
| READ_POLE | 4 (0.7) | 0 (0.0) | 3 (1.0) | 1 (1.0) |
| Neoplasm_Disease_Stage (%) |  |  |  |  |
| Missing | 12 (2.1) | 5 (3.1) | 4 (1.3) | 3 (3.1) |
| STAGE I | 100 (17.5) | 34 (21.4) | 47 (15.0) | 19 (19.6) |
| STAGE II | 211 (37.0) | 63 (39.6) | 118 (37.6) | 30 (30.9) |
| STAGE III | 166 (29.1) | 36 (22.6) | 100 (31.8) | 30 (30.9) |
| STAGE IV | 81 (14.2) | 21 (13.2) | 45 (14.3) | 15 (15.5) |

| Variable | Exp (coef) | Exp (-coef) | Lower .95 | Upper .95 |
| --- | --- | --- | --- | --- |
| *N4likevirus* | 7.378 e+03 | 1.355 e-04 | 1.835 e-07 | 2.967 e+14 |
| *Ambidensovirus* | 3.004 e+03 | 3.329 e-04 | 1.051 e-02 | 8.582 e+08 |
| *Synechococcus* | 3.885 e+02 | 2.574 e-03 | 2.372 e-04 | 6.362 e+08 |
| *Thermithiobacillus* | 5.165 e-05 | 1.936 e+04 | 7.092 e-13 | 3.761 e+03 |
| *Hydrocarboniphaga* | 1.073 e-04 | 9.318 e+03 | 1.689 e-10 | 6.819 e+01 |
| *Rhodovibrio* | 9.108 e-04 | 1.098 e+03 | 7.268 e-13 | 1.141 e+06 |
| *Gloeobacter* | 2.278 e-04 | 4.389 e+03 | 4.656 e-11 | 1.115 e+03 |
| *Candidatus Nitrosotenuis* | 1.266 e+04 | 7.901 e-05 | 3.479 e-04 | 4.603 e+11 |
| Diagnosis age | 9.862 e-01 | 1.014 e+00 | 9.199 e-01 | 1.057 e+00 |
| Buffa hypoxia score | 1.063 e+00 | 9.406 e-01 | 9.351 e-01 | 1.209 e+00 |
| Disease free months | 7.797 e-01 | 1.283 e+00 | 6.572 e-01 | 9.249 e-01 |
| TMB | 9.999 e-01 | 1.000 e+00 | 9.856 e-01 | 1.015 e+00 |
| Patient Weight | 9.215 e-01 | 1.085 e+00 | 8.177 e-01 | 1.038 e+00 |

**Supplementary Table 4:** The results from the Cox regression show the hazard ratios of the significant OTUs and clinical features. The hazard ratios were varied for each OTUs, as illustrated by a large 95% confidence interval. *Ambidensovirus* had consistently high hazard ratios whilst *Hydrocarboniphaga* was the OTU with the most consistently low hazard ratio. Furthermore, disease-free months and patient weight had reduced hazard ratios.

**Supplementary Table 5:** The clustering parameters show the clustering efficiency of each of the four clustering methods. K-means has high Calinski Harabasz, connectivity and SD, but K-means was mostly chosen on visual inspection of the clusters.

| Algorithms | Calinski Harabasz | Dunn | Gamma | C-index | Davies Bouldin | SD | S dbw | Silhouette | Compactness | Connectivity |
| --- | --- | --- | --- | --- | --- | --- | --- | --- | --- | --- |
| HC Euclidean | 3.983 | 0.696 | 0.969 | 0.092 | 0.471 | 1.6 | Inf | 0.377 | 2.214 | 5.858 |
| PAM Euclidean | 6.365 | 0.096 | -0.049 | 0.513 | 6.319 | 7.1 | Inf | -0.077 | 2.196 | 475.157 |
| KM | 10.967 | 0.248 | 0.529 | 0.238 | 4.466 | 8.2 | Inf | 0.122 | 2.182 | 259.519 |
| C-MEANS | 11.992 | 0.191 | 0.289 | 0.339 | 4.719 | 8.2 | Inf | 0.05 | 2.176 | 387.637 |

**Supplementary Table 6:** All the R packages and corresponding version of the packages are listed here.

| Methods | R Packages used | Version |
| --- | --- | --- |
| Baseline table | *tableone* | 0.13.2 |
| Pre-processing | *dplyr* | 1.0.8 |
|  | *ggplot2* | 3.3.5 |
| Clustering | *factoextra* | 1.0.7 |
|  | *cluster* | 2.1.3 |
|  | *clustree* | 0.5.0 |
|  | *diceR* | 2.0.0 |
|  | *stats* | 4.2.0 |
|  | *vegan* | 2.6.2 |
| Diversity analysis | *vegan* | 2.6.2 |
|  | *ggplot2* | 3.3.5 |
| Feature selection | *rstatix* | 0.7.0 |
|  | *randomForest* | 4.7.1 |
|  | *varSelRF* | 0.7.8 |
|  | *glmnet* | 4.1.4 |
|  | *caret* | 6.0.92 |
| Boxplot | *ggplot2* | 3.3.5 |
|  | *gridextra* | 2.3 |
| Validation | *curedMetagenomicData* |  |
|  | *sva* |  |
| Survival | *survival* | 3.3.1 |
|  | *survminer* | 0.4.9 |
|  | *ggplot2* | 3.3.5 |
|  | *gridextra* | 2.3 |
|  | *dplyr* | 1.0.8 |
